# Urinary Uromodulin for Distinguishing Between Glomerulonephritis, Diabetic Kidney Disease, and Nephrosclerosis in a Biopsy-Proven Cohort

**DOI:** 10.3390/diagnostics16172741

**Published:** 2026-08-26

**Authors:** Tomoaki Takata, Yukari Mae, Kensuke Kawata, Yudai Fujino, Sosuke Taniguchi, Takuji Iyama, Shotaro Hoi, Makoto Tahira, Hajime Isomoto

**Affiliations:** 1 Division of Gastroenterology and Nephrology, Faculty of Medicine, Tottori University, Yonago 683-8504, Japan; 2 Advanced Medicine, Innovation and Clinical Research Center, Tottori University Hospital, Yonago 683-8504, Japan

**Keywords:** chronic kidney disease, glomerulonephritis, diabetic kidney disease, nephrosclerosis, renal biopsy, urinary biomarker, UMOD, uromodulin

## Abstract

**Background/Objectives**: Distinguishing glomerulonephritis (GN) from diabetic kidney disease (DKD) and nephrosclerosis (NS) is clinically important. Urinary uromodulin (uUMOD), a biomarker of renal tubular integrity, may help discriminate among these chronic kidney disease (CKD) etiologies. We evaluated the diagnostic utility of uUMOD for differentiating GN from DKD and NS. **Methods**: This retrospective single-center study included 90 patients with biopsy-proven GN (*n* = 30), DKD (*n* = 25), or NS (*n* = 35). First-morning spot urine samples obtained before biopsy were analyzed for uUMOD concentration and normalized to urinary creatinine levels (uUMOD/Cr). **Results**: The uUMOD/Cr ratio was significantly higher in GN (21.2 mg/gCr [10.3–31.3]) than in DKD (5.5 [3.6–12.3], *p* < 0.001) or NS (10.7 [6.4–19.9], *p* = 0.021). In multivariate analyses adjusted for age, sex, estimated glomerular filtration rate, interstitial fibrosis, and urinary protein, the uUMOD/Cr ratio was independently associated with GN (*p* = 0.003) and this correlation remained statistically significant after additional adjustment for diabetes mellitus. Receiver operating characteristic curve analysis showed an area under the curve value of 0.833 for GN versus DKD, 0.714 for GN versus NS, and 0.764 for GN versus DKD and NS combined. A cutoff value of 16.5 mg/gCr yielded 72.8% sensitivity and 70.0% specificity for identifying GN. **Conclusions**: uUMOD secretion is preserved in GN compared to DKD or NS and shows moderate diagnostic utility for differentiating between these biopsy-proven CKD etiologies. uUMOD may serve as a simple, noninvasive biomarker when biopsy is difficult or clinical findings are inconclusive.

## 1. Introduction

Chronic kidney disease (CKD) is a major global health concern because of its increasing prevalence, association with cardiovascular disease, and frequent progression to end-stage renal disease [1,2,3]. Regardless of the initiating insult, progressive CKD ultimately leads to loss of functional nephron mass, accumulation of metabolic abnormalities, and a substantial decline in quality of life. Among the many causes of CKD, diabetic kidney disease (DKD), glomerulonephritis (GN), and nephrosclerosis (NS) account for a large proportion of patients who eventually require renal replacement therapy [4,5]. Importantly, these three diseases represent distinct pathological processes with different dominant compartments of injury, different treatment strategies, and different clinical implications.

An accurate etiological diagnosis is therefore essential in routine nephrology practice. GN is often driven by immune-mediated glomerular injury and may require immunosuppressive therapy, close monitoring of urinary sediment, and disease-specific evaluation. Conversely, DKD and NS are generally managed with optimization of metabolic and hemodynamic risk factors, including strict blood pressure control, treatment of hyperglycemia, management of dyslipidemia and hyperuricemia, and use of renoprotective agents [6,7,8,9]. Distinguishing GN from DKD or NS is clinically important because treatment decisions differ substantially across these conditions. However, distinguishing between DKD, NS, and GN is often challenging. Proteinuria, hematuria, reduced kidney function, and hypertension are common to many kidney diseases and often overlap across etiologies. The diagnostic challenge is particularly evident in patients with diabetes or longstanding hypertension; heavy proteinuria in a patient with diabetes does not necessarily indicate DKD, and microscopic hematuria does not always imply primary GN. Similarly, age-related vascular changes and chronic nephrosclerosis can coexist with glomerular disease. Thus, although clinical clues such as duration of diabetes, diabetic retinopathy, urinary sediment findings, and the pattern of renal function decline are informative, they are not sufficiently specific to allow reliable etiological discrimination in all cases.

The consequences of etiologic misclassification are not merely academic. Labeling a treatable immune-mediated GN as DKD or NS may delay immunosuppressive therapy and allow potentially reversible injury to progress, whereas attributing kidney disease in a person with diabetes solely to DKD may overlook a coexisting or alternative glomerular disease that would respond to specific treatment. Non-diabetic kidney disease is a well-recognized finding when patients with diabetes undergo biopsy, and clinical algorithms based on the duration of diabetes, the presence of retinopathy, and the degree of proteinuria identify only a proportion of such cases. The diagnosis of hypertensive nephrosclerosis is likewise often made by exclusion in older patients with vascular risk factors, even though biopsy can reveal an unsuspected glomerular or interstitial process. Because the decision to perform a biopsy must weigh these diagnostic uncertainties against procedural risk [10], a noninvasive test that shifts the pre-test probability toward or away from a glomerular process would be clinically valuable, particularly when it can be obtained from a routine spot urine sample. These considerations motivated us to examine whether a kidney specific protein could contribute such information.

Renal biopsy remains the gold standard for establishing the histopathological diagnosis of kidney diseases. It provides direct information on glomerular, tubular, interstitial, and vascular lesions and is therefore indispensable when a precise diagnosis is required. However, renal biopsy is invasive and carries a risk of bleeding and other complications [10]. Renal biopsy is not performed in some cases because of contraindications, patient preference, or limited expected benefit. Therefore, there is a clear clinical need for noninvasive biomarkers that can complement routine clinical information and help estimate the underlying CKD etiology. Most conventional renal biomarkers provide limited etiologic information [11]. Serum creatinine and estimated glomerular filtration rate (eGFR) reflect overall filtration but do not identify the primary site or mechanism of injury [12]. Urinalysis, including proteinuria and hematuria, is a marker of glomerular dysfunction, but lacks sufficient sensitivity and specificity for distinguishing CKD etiologies. A biomarker that reflects a different structural compartment of the kidney may therefore provide complementary information beyond glomerular injury.

Urinary biomarkers of renal tubular integrity are particularly attractive because many CKD etiologies differ not only in the severity of glomerular injury but also in the extent and distribution of tubulointerstitial injury [13,14,15,16]. Uromodulin (UMOD), also known as Tamm-Horsfall protein, is a kidney-specific glycoprotein produced exclusively by the epithelial cells of the thick ascending limb of the loop of Henle and the early distal convoluted tubule, and it is the most abundant protein in normal human urine [17,18]. uUMOD has emerged as a candidate biomarker reflecting renal tubular integrity because urinary excretion of UMOD depends on the functional capacity of the nephron [18,19,20]. uUMOD may help discriminate among these major CKD etiologies because GN primarily involves glomerular damage, whereas DKD and NS are often accompanied by more prominent tubulointerstitial injury.

At the molecular level, UMOD is encoded by the UMOD gene and is targeted to the apical membrane of tubular epithelial cells, where most of the protein is released into the tubular lumen [17,18]. Because UMOD is synthesized by a defined segment of each functioning nephron, its urinary excretion has been proposed to reflect the number of intact nephrons and the structural integrity of the distal tubule rather than glomerular filtration alone [19,20]. Consistent with this concept, lower uUMOD concentrations have been linked to reduced nephron mass, a faster decline in eGFR, and a higher risk of progressive kidney disease [20]. However, most previous studies evaluated uUMOD in relation to kidney function or prognosis in mixed or clinically defined cohorts, and few have directly compared uUMOD among the major histological categories of CKD within a single biopsy-proven population. Whether uUMOD can help distinguish GN, a predominantly glomerular disease, from DKD and NS, in which tubulointerstitial and vascular injury are typically more prominent, therefore remains insufficiently characterized. We addressed this gap by measuring uUMOD in patients with biopsy-proven GN, DKD, or NS, and by assessing whether uUMOD concentrations differ among these etiologies and provide diagnostic value for identifying GN.

## 2. Materials and Methods

### 2.1. Study Population

This retrospective study included patients who underwent renal biopsy between June 2018 and December 2023 at Tottori University Hospital, Japan. The indications for renal biopsy and the biopsy procedure have been described previously [21]. In brief, renal biopsy was indicated for patients with persistent hematuria and/or proteinuria, proteinuria > 0.5 g/day, a rapid decline in renal function, or gross hematuria. Histopathological diagnoses were established by an experienced nephrologist according to standard criteria. Patients with a biopsy-proven diagnosis of GN, DKD, or NS were included in the analysis. Clinical and laboratory data were collected from electronic medical records. This study was conducted in accordance with the principles of the Declaration of Helsinki and was approved by the Research Ethics Committee of Tottori University Hospital (Ethical Approval Number: 23A079).

### 2.2. Measurement of uUMOD

First-morning spot urine samples were collected on the day of biopsy, prior to the procedure, and stored at −80 °C until analysis and repeated freeze–thaw cycles were avoided. uUMOD concentrations were measured using a commercially available human UMOD enzyme-linked immunosorbent assay kit (BioVendor, Brno, Czech Republic), according to the protocol of the manufacturer. The analytical sensitivity, detection range, and intra- and inter- assay coefficients of variation were based on the manufacturer’s validation data. All samples were measured in duplicate, and the mean value was used. Calibration was performed using standard curves generated from the manufacturer’s standards on each plate. The assay procedure was performed without access to clinical and pathological information. To correct for variations in urine concentration, the uUMOD concentration was normalized to urinary creatinine, and the urinary UMOD-to-creatinine ratio (uUMOD/Cr) was used for analysis.

### 2.3. Histological Evaluation

Histological diagnoses were determined based on kidney biopsy findings assessed by experienced nephrologists and renal pathologists using light microscopy, immunofluorescence, and electron microscopy when available. GN was classified based on the presence of primary glomerular lesions consistent with specific glomerular diseases. DKD was diagnosed based on a characteristic diabetic glomerular lesion. NS was defined by predominant chronic vascular and ischemic changes, including arteriosclerosis, arteriolar hyalinosis, and chronic tubulointerstitial injury, without evidence of primary glomerular disease. Interstitial fibrosis and tubular atrophy (IFTA) were evaluated using periodic acid–methenamine silver-stained kidney biopsy specimens. The extent of chronic tubulointerstital injury was graded semi-quantitatively according to the percentage of area affected by interstitial fibrosis and tubular atrophy; score 0, absent or <10%; score 1, 10–25%; score 2, 26–50%; and score 3, >50%.

### 2.4. Statistical Analysis

Continuous variables were expressed as mean ± standard deviation or median (interquartile range). Distribution of the variables were assessed using the Shapiro–Wilk test. Variables with a normal distribution are presented as mean ± standard deviation and compared using parametric tests. Variables with non-normal distributions are presented as median (interquartile range) and were analyzed using nonparametric tests. For comparisons among the three groups, one-way analysis of variance followed by Tukey’s post hoc test was used for normally distributed variables, whereas the Kruskal–Wallis test followed by Dunn’s test with Bonferroni correction was used for non-normally distributed variables. The correlations between two variables were analyzed using Pearson’s correlation coefficient for normally distributed variables or the Spearman rank correlation coefficient for non-normally distributed variables. Multiple logistic regression analyses, in which age, sex, eGFR, urinary protein, and the uUMOD/Cr were included as explanation variables, were carried out to identify factors that influenced the histological diagnosis. The diagnostic ability of uUMOD/Cr was assessed by employing receiver operating characteristic (ROC) curves. Statistical analyses were performed using StatFlex software version 7 for Windows (Artec, Osaka, Japan). A two-tailed *p*-value < 0.05 was considered statistically significant.

## 3. Results

### 3.1. Patient Characteristics

A total of 90 patients were included in the final analysis, comprising 30 patients with GN, 25 with DKD, and 35 with NS. Among the 30 patients with GN, the pathological subtypes were as follows: IgA nephropathy (*n* = 14), membranous nephropathy (*n* = 4), microscopic polyangiitis (*n* = 3), focal segmental glomerulosclerosis (*n* = 2), minimal change disease (*n* = 2), and other (*n* = 5). The baseline characteristics of the study population are summarized in Table 1. Significant differences were observed among the three groups in age, sex, eGFR, IFTA score, and urinary protein excretion. Patients in the GN group were the youngest with a mean age of 49.9 ± 23.3 years, whereas those in the NS group were the oldest with a mean age of 64.7 ± 14.2 years (*p* = 0.003). The proportion of men also differed among the groups (*p* = 0.017), with men predominating in the DKD and NS groups compared with the GN group. Kidney function assessed by eGFR values was significantly better in the GN group than the DKD and NS groups. The mean eGFR value was 68.4 ± 31.9 mL/min/1.73 m^2^ in the GN group, compared with 46.3 ± 24.4 mL/min/1.73 m^2^ in the DKD and 53.2 ± 23.3 mL/min/1.73 m^2^ in the NS group (*p* = 0.009). Urinary protein excretion also differed significantly among the groups (*p* < 0.001). The urinary protein-to-creatinine ratio (UPCR) was highest in the DKD group, followed by NS and GN groups.

### 3.2. Comparison of uUMOD/Cr Levels Among Groups

**Figure 1:**
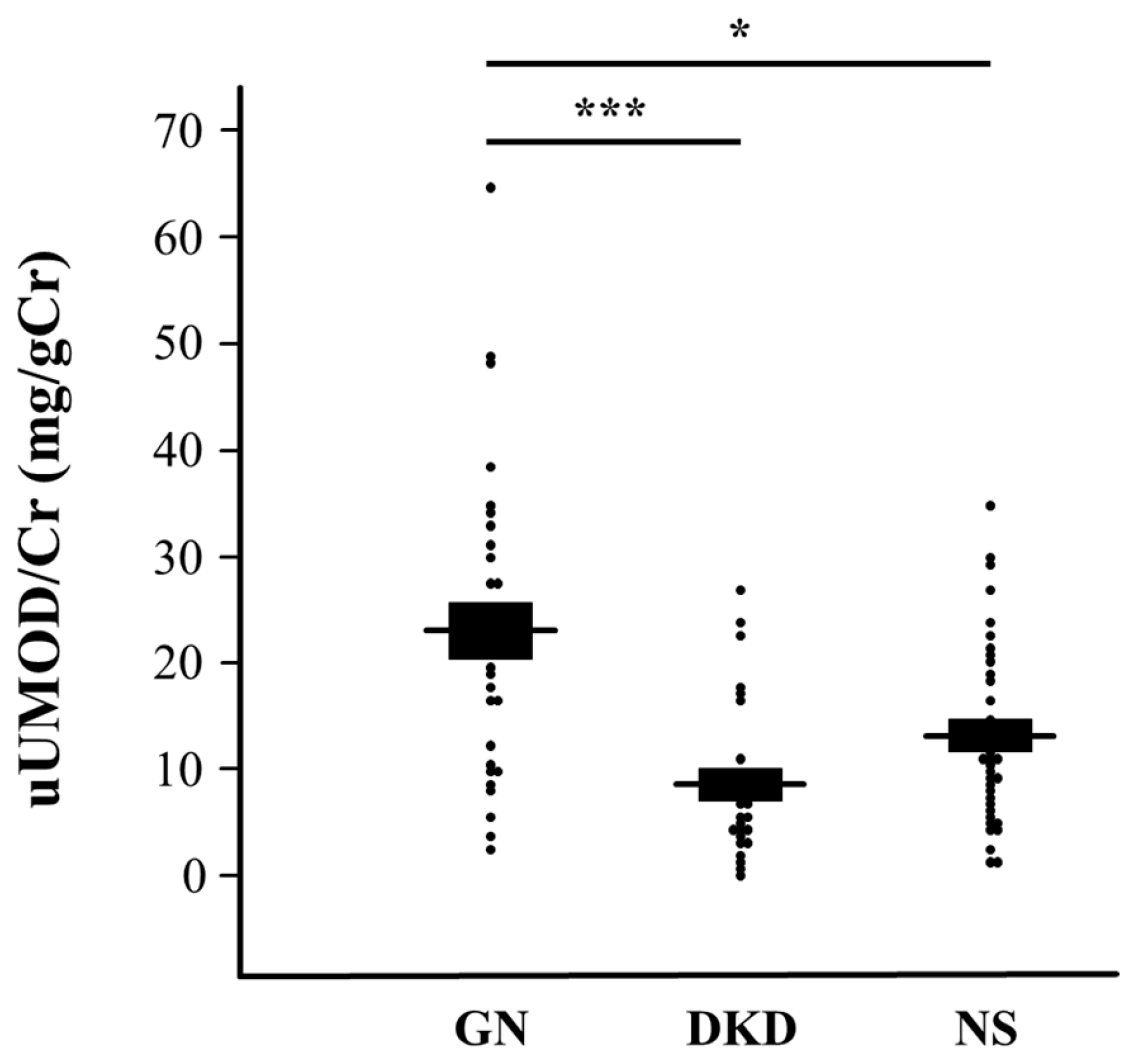
Comparison of urinary uromodulin (uUMOD) levels among patients with GN, DKD, and NS. The urinary UMOD-to-creatinine (uUMOD/Cr) ratio was measured in first-morning spot urine samples obtained on the day of renal biopsy. Each dot represents an individual patient, and horizontal bars indicate the median and interquartile ranges. The uUMOD/Cr ratio was significantly higher in the GN than the DKD (*** *p* < 0.001) and NS groups (* *p* < 0.05). The Kruskal–Wallis test with a *post hoc* Dunn test was employed.

We compared the magnitude of the uUMOD/Cr ratio across the three groups. The GN group showed significantly higher urinary excretion of UMOD than both the DKD and NS groups. Median uUMOD/Cr values were 21.2 (10.3–31.3) mg/gCr in the GN group, 5.5 (3.6–12.3) mg/gCr in the DKD group, and 10.7 (6.4–19.9) mg/gCr in the NS group. The difference between GN and DKD groups was highly significant (*p* < 0.001), and the difference between GN and NS groups was also significant (*p* = 0.021) (Figure 1).

### 3.3. Diagnostic Performance of uUMOD

**Figure 2:**
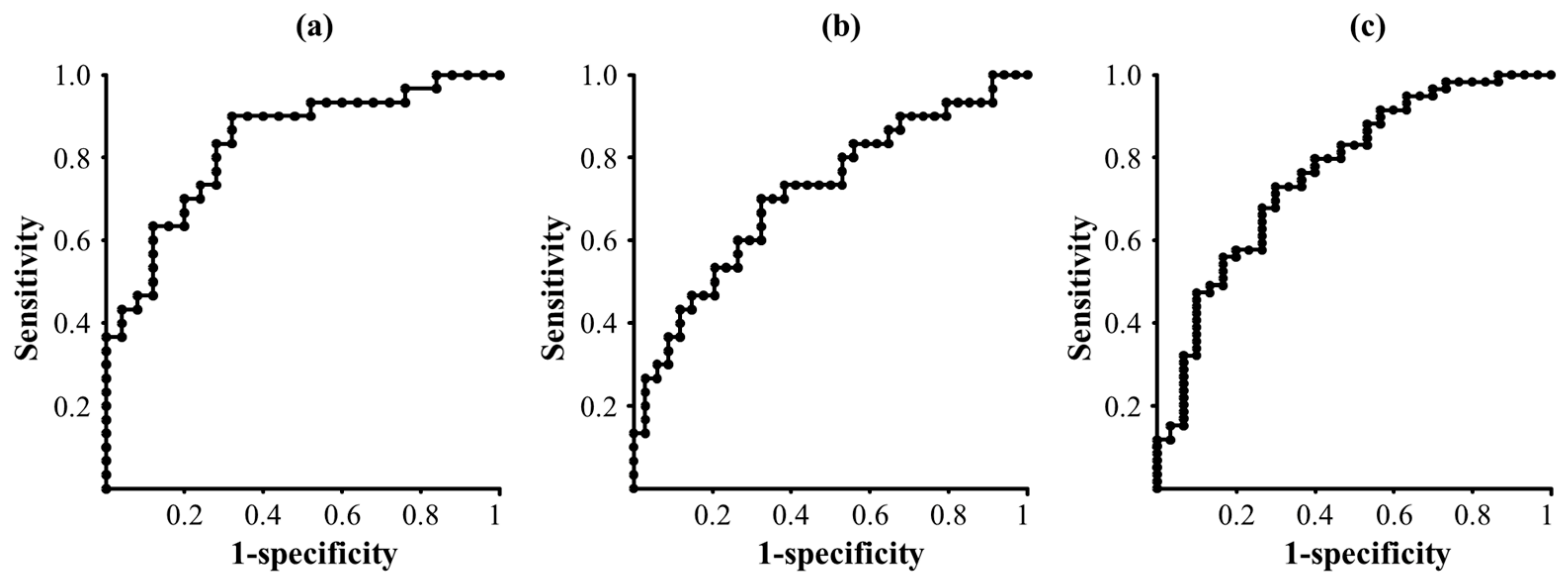
Receiver operating characteristic (ROC) curves for the urinary uromodulin-to-creatinine ratio in the differential diagnosis of glomerulonephritis (GN). Discrimination of GN from (**a**) diabetic kidney disease (DKD; area under the curve [AUC] = 0.833), (**b**) nephrosclerosis (NS; AUC = 0.714), and (**c**) DKD and NS combined (AUC = 0.764).

To evaluate the potential clinical usefulness of uUMOD concentration as a discriminatory biomarker, we performed ROC curve analyses. The AUC value for differentiating GN from DKD was 0.833. The AUC value for differentiating GN from NS was 0.714. When GN was compared with both DKD and NS combined, the AUC value was 0.764 (Figure 2). Using a cut off value of 16.5 mg/gCr, the uUMOD/Cr ratio achieved a sensitivity of 72.8% and a specificity of 70.0% for identifying GN.

### 3.4. Correlation of uUMOD with Clinical Parameters

To better understand the determinants of uUMOD excretion, we examined correlations between the uUMOD/Cr ratio and key clinical variables. Univariate analysis showed that the uUMOD/Cr ratio was significantly associated with several indices of kidney injury and renal function. Specifically, the uUMOD/Cr parameter correlated negatively with serum creatinine (r = −0.472, *p* < 0.001), urinary protein excretion (r = −0.376, *p* < 0.001), and IFTA score (r = −0.335, *p* = 0.001), and positively with eGFR (r = 0.446, *p* < 0.001). Conversely, no significant correlations were observed between the uUMOD/Cr ratio and age, height, body weight, or body mass index.

**Figure 3:**
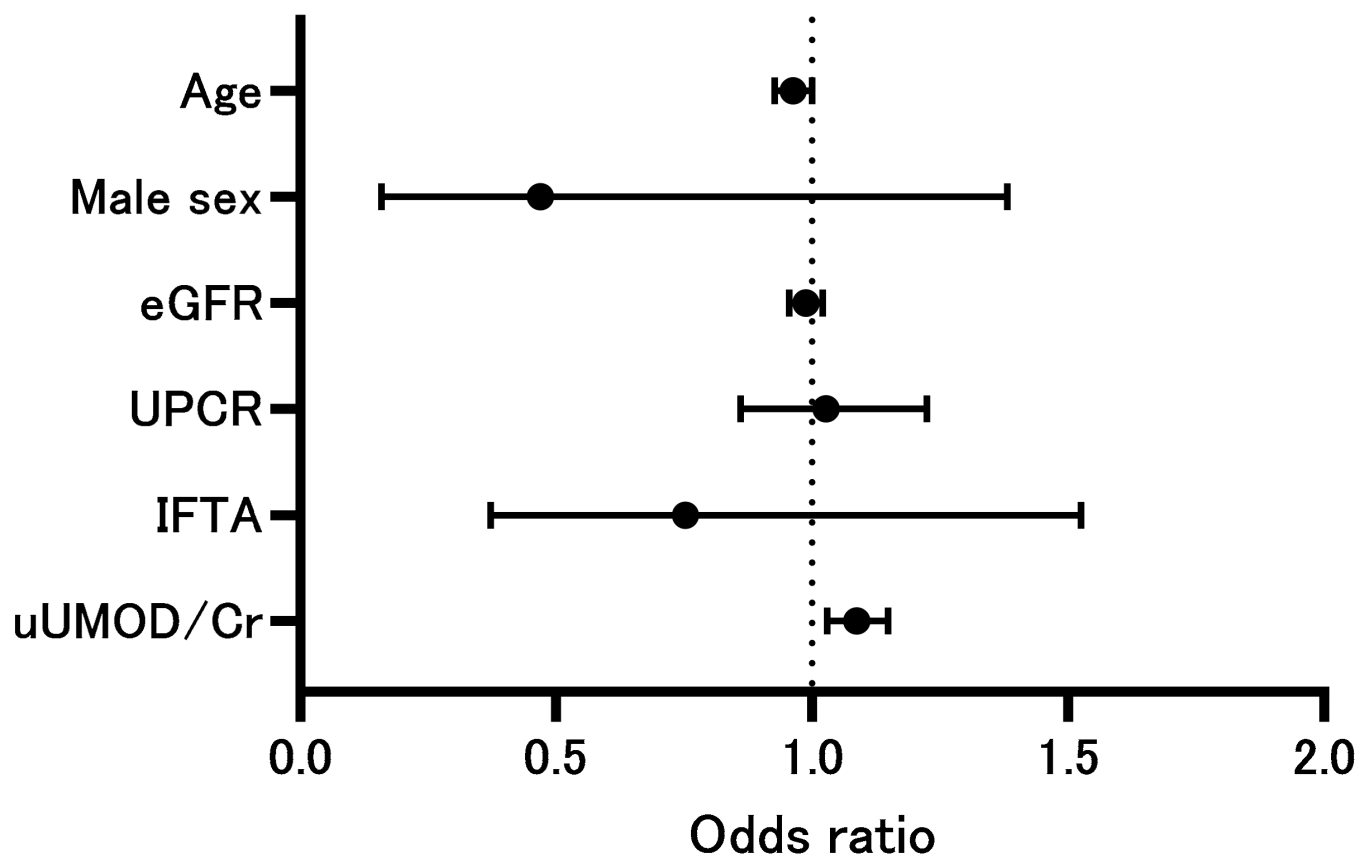
Multivariate logistic regression analysis for the histological diagnosis of GN. Forest plot showing odds ratios and 95% confidence intervals for age, male sex, urinary protein-to-creatinine ratio (UPCR), estimated glomerular filtration rate (eGFR), and urinary uromodulin-to-creatinine ratio (uUMOD/Cr) in relation to the diagnosis of GN versus DKD/NS. The vertical dashed line indicates an odds ratio of 1.0. Higher values of the uUMOD/Cr ratio were independently associated with GN after adjustment for the other covariates.

We performed multivariate analysis to determine whether the association between uUMOD/Cr and GN was independent of eGFR and proteinuria because these two parameters differed among the three groups and were also associated with the uUMOD/Cr ratio. A multivariate logistic regression model, including age, sex, eGFR, urinary protein, IFTA score and uUMOD/Cr as explanatory variables, showed that the uUMOD/Cr ratio remained an independent predictor of GN (*p* = 0.003) (Table 2 and Figure 3). We adjusted for the presence of diabetes mellitus because diabetes itself could have influenced uUMOD excretion and may have confounded the distinction between GN and DKD. Even after this adjustment, the association between uUMOD/Cr and GN remained statistically significant (*p* < 0.05). Because uUMOD/Cr and urinary protein excretion showed skewed distributions, additional analyses were performed using log-transformed values. In multivariable logistic regression analysis incorporating uUMOD/Cr and log-transformed UPCR, log-transformed uUMOD/Cr remained independently associated with GN after adjustment for the other factors.

## 4. Discussion

In this study, we examined uUMOD concentration in a biopsy-based cohort of patients with three major causes of CKD. We found that the uUMOD/Cr ratio was significantly higher in patients with GN than in those with DKD or NS. We also found that the uUMOD/Cr ratio was associated with serum creatinine, eGFR, and urinary protein excretion, indicating that it reflects the severity of renal injury and loss of renal function. ROC analysis showed that the uUMOD/Cr ratio had moderate-to-good ability to distinguish GN from DKD and NS. Finally, multivariate analysis confirmed that uUMOD/Cr was independently associated with the diagnosis of GN, supporting its potential discriminatory value as a noninvasive biomarker for the diagnostic evaluation of CKD, particularly in settings where renal biopsy is limited or contraindicated.

We observed that uUMOD concentration was relatively higher in patients with GN compared with those with DKD or NS. These findings are biologically plausible because GN is primarily a disease of the glomerulus, particularly in its earlier or more active phases, and may therefore leave the distal nephron relatively preserved compared with diseases that diffusely affect the tubulointerstitial and vascular compartments. In such settings, the capacity to synthesize and secrete uromodulin may remain relatively intact despite the presence of substantial glomerular injury. This interpretation is also compatible with prior observations in patients with immunoglobulin A (IgA) nephropathy and other glomerular diseases, in whom lower uUMOD concentration has been associated with more advanced chronic tubulointerstitial damage [22]. Glomerular disease *per se* may not reduce uUMOD levels substantially until secondary renal tubular and interstitial injury develops. Therefore, relatively higher uUMOD concentrations in GN may reflect preservation of distal tubular integrity rather than absence of renal injury.

The lower uUMOD/Cr ratio observed in DKD is also consistent with the current understanding of diabetic kidney injury. Although DKD has historically been conceptualized as a glomerular disease characterized by mesangial expansion and glomerular basement membrane thickening, it is also recognized that renal tubular and interstitial injury play central roles in its pathogenesis and progression [8,23,24]. Chronic hyperglycemia, oxidative stress, altered intrarenal hemodynamics, inflammation, and profibrotic signaling all contribute to renal tubular dysfunction and interstitial fibrosis. In the early stages of diabetes, glomerular hyperfiltration and compensatory changes in renal tubules may maintain or even transiently increase some aspects of renal tubular function. However, as the disease progresses, structural damage accumulates and renal tubular reserve declines [25,26]. Reduced uUMOD excretion in DKD may therefore reflect a progressive loss of renal tubular integrity and functioning nephron mass. In addition, a large population-based study reported a negative association between uUMOD levels and diabetes [27]. From a clinical perspective, this makes uUMOD levels particularly interesting because these may capture a dimension of DKD severity that is not fully described by albuminuria alone.

Similarly, the reduction in uUMOD/Cr in NS can be explained by the dominant pathological mechanisms of nephrosclerotic kidney disease. NS is characterized by chronic vascular injury, atherosclerosis, ischemic nephron loss, tubular atrophy, and interstitial fibrosis [23,24]. Chronic ischemic and vascular injury may impair the epithelial cells of renal tubules responsible for UMOD production because the renal medulla and distal nephron are highly dependent on intact perfusion and oxygen delivery. Thus, the comparatively reduced uUMOD levels in NS are consistent with widespread chronic tubulointerstitial and vascular remodeling. The fact that the AUC value for GN versus NS was lower than that for GN versus DKD likely reflects the biological heterogeneity of NS and the frequent coexistence of glomerular sclerosis and chronic interstitial damage across disease categories.

The interpretation of uUMOD as an etiology-related biomarker is supported by the biological functions of uromodulin. Beyond being a passive byproduct of tubular metabolism, uromodulin participates in several homeostatic processes, including modulation of sodium and water handling in the thick ascending limb, protection against urinary tract infection and nephrolithiasis, and regulation of innate immune and inflammatory responses within the kidney [17,18,19]. These functions depend on an adequate mass of healthy distal tubular epithelium, so conditions that injure this compartment are expected to reduce uromodulin synthesis. Experimental and clinical data indicate that uromodulin production per functioning nephron is tightly regulated and that its excretion declines in parallel with tubular atrophy and interstitial fibrosis [17,20]. In GN, where injury is initially concentrated in the glomerulus, a relatively larger proportion of distal tubular mass may remain functional, whereas in DKD and NS the distal nephron is exposed to chronic metabolic, hemodynamic, and ischemic stress that diffusely compromises tubular function. Viewed in this light, the differences in uUMOD that we observed are not simply a consequence of differences in glomerular filtration but reflect the disease-specific topography of nephron injury, which is precisely the information that conventional glomerular markers fail to convey. Interestingly, in multivariable analysis including the IFTA score, uUMOD/Cr remained independently associated with GN. This finding suggests that the difference in uUMOD among CKD etiologies cannot be explained solely by the severity of chronic tubulointerstitial injury. Rather, uUMOD may capture additional biological information related to the predominant compartment of renal injury and the preservation of functional tubular mass.

Our findings also help to position uUMOD among the broader panel of urinary biomarkers proposed for CKD. Tubular injury markers such as neutrophil gelatinase-associated lipocalin (NGAL), kidney injury molecule-1 (KIM-1), liver-type fatty acid-binding protein (L-FABP), and N-acetyl-β-D-glucosaminidase are typically up-regulated in response to active or acute tubular stress and tend to rise with injury [13,14,15]. In contrast, uUMOD behaves as a reserve marker; it is constitutively produced by intact distal tubular epithelium, so its excretion falls as functioning nephron mass and tubular integrity are lost [17,18]. This conceptual difference is clinically relevant, because a marker that decreases with cumulative structural damage may capture a different and complementary dimension of disease from markers that increase with active injury. In the present study, the relative preservation of uUMOD in GN, despite considerable glomerular injury, and its reduction in DKD and NS are consistent with this concept and suggest that uUMOD reflects the distribution of injury between the glomerular and tubulointerstitial compartments rather than its mere presence. Combining a reserve marker such as uUMOD with injury markers and with glomerular markers such as albuminuria and proteinuria may therefore provide a more complete picture of the underlying disease process than any single marker alone.

An important aspect of our results is the relationship between the uUMOD/Cr ratio and conventional markers of kidney function. As expected, the uUMOD/Cr ratio correlated positively with eGFR values and negatively with serum creatinine and urinary protein levels. These associations indicate that uUMOD levels are influenced by overall renal functional status and disease burden. However, our critical observation in this study is that the uUMOD/Cr ratio remained independently associated with GN after adjustment for these parameters. This suggests that uUMOD levels provide more than a simple readout of kidney function. Although our findings indicate that uUMOD concentration provides complementary information to aid etiological diagnosis, it should not be interpreted as meaning that uUMOD levels are universally high in all forms of GN. Advanced GN can lead to extensive interstitial fibrosis, tubular atrophy, and nephron loss, all of which are expected to reduce uUMOD excretion. Therefore, the discriminatory ability of uUMOD concentration is likely to depend on disease stage and chronicity. The uUMOD parameter may be best understood as a biomarker that reflects the balance between disease-specific pathophysiology and the cumulative burden of tubulointerstitial damage.

The diagnostic role of uUMOD examined here may also carry prognostic implications. Low uUMOD concentrations have repeatedly been associated with a faster decline in eGFR, incident CKD, and adverse cardiovascular and mortality outcomes in both general and CKD populations, reflecting the progressive loss of functioning nephron mass [20,27]. If the lower uUMOD levels observed in DKD and NS in our cohort track with a greater burden of irreversible tubulointerstitial damage, uUMOD could in principle convey information about both the type and the likely trajectory of disease from a single measurement. This dual diagnostic and prognostic potential distinguishes uUMOD from markers that capture only the current level of kidney function, and it strengthens the rationale for evaluating uUMOD in longitudinal studies that record hard renal endpoints. Whether the etiologic differences we identified translate into differences in outcome within each disease category remains to be tested directly, and prospective follow-up of biopsy-proven cohorts with serial uUMOD measurement would be the most direct way to address this question.

In clinical practice, nephrologists frequently encounter patients in whom the distinction between GN and DKD or NS is uncertain. A patient with longstanding diabetes may develop extensive proteinuria and display declining kidney function but may also exhibit hematuria, raising the possibility of superimposed GN. Conversely, an older patient with hypertension and moderate proteinuria may have NS, but a treatable glomerular process cannot always be excluded on the basis of clinical findings alone. In such situations, a renal biopsy is often considered, but the decision can be difficult when procedural risks are high or when the likelihood of changing disease management is unclear [11,28]. A noninvasive biomarker such as uUMOD, may provide complementary information and help refine pre-biopsy probability, support risk stratification, and inform diagnostic strategy. Using uUMOD has advantages in terms of feasibility and patient burden because it can be measured from a spot urine sample and normalized to urinary creatinine.

Another clinically meaningful observation is that the diagnostic performance of the uUMOD/Cr ratio in our cohort was comparable to that of traditional clinical clues often used to infer etiology. Markers such as diabetic retinopathy and microscopic hematuria may be helpful in part. In a meta-analysis of 45 studies including 4561 patients with diabetes, the presence of diabetic retinopathy showed a sensitivity of only 67% and specificity of 78% for the diagnosis of DKD [29]. Similarly, in a biopsy-based analysis of 51 patients with insulin independent diabetes mellitus, the presence of microscopic hematuria demonstrated a sensitivity of 59% and specificity of 68% for the diagnosis of GN [30]. In our study, the magnitude of the uUMOD/Cr ratio achieved a sensitivity of 72.8% and a specificity of 70.0% for identifying GN. These figures indicate that uUMOD levels are not definitive on their own, but they compare favorably with common bedside indicators and represent meaningful diagnostic value. It is possible that combining uUMOD concentration with other conventional variables such as age, presence of diabetes mellitus, blood pressure, urinary sediment findings, and albuminuria, would further improve discriminatory performance. The development of such integrated diagnostic models is an important direction for future research.

This study has several limitations. First, this was a single-center retrospective study with a relatively modest sample size, and the findings may not be generalizable to broader populations. Selection bias is possible because all included patients underwent biopsy, implying that our cohort may not fully represent typical patients with clearly diagnosed DKD or NS who are managed without biopsy. Second, the GN group likely included multiple pathological subtypes with different degrees of chronicity and tubulointerstitial involvement. We were unable to analyze each GN subtype separately because of the small sample size. Such disease-specific analysis may be desirable because active proliferative GN, membranous nephropathy, and IgA nephropathy may have different uUMOD profiles. Third, eGFR differed across groups, and although multivariate adjustment was performed, residual confounding related to kidney function or nephron mass cannot be completely excluded. Another limitation is that our study focused on diagnostic discrimination rather than direct correlation with histological severity scores such as renal tubular atrophy, interstitial fibrosis, or vascular lesions. Future studies that link uUMOD levels quantitatively to histological lesions would provide important mechanistic insight because UMOD levels reflect distal renal tubular integrity and tubulointerstitial reserve. However, our study also has strengths such as the fact that the diagnoses were biopsy-proven and reduced misclassification and that the urine samples were collected on the day of biopsy, closely aligning biomarker measurement with histological assessment. We also included three clinically important CKD etiologies that are common in nephrology practice and often difficult to distinguish based on clinical findings alone.

## 5. Conclusions

In conclusion, the uUMOD/Cr ratio was significantly higher in patients with GN than in those with DKD or NS in a biopsy-proven CKD cohort. Higher uUMOD/Cr values remained independently associated with GN after adjustment for conventional clinical variables. These findings support the concept that uUMOD concentration reflects renal tubular integrity and captures disease-specific differences in the balance between glomerular and tubulointerstitial injury. As a simple and noninvasive biomarker, uUMOD may be a useful adjunct in the etiological evaluation of CKD, particularly when renal biopsy is difficult to perform or when clinical findings alone are insufficient to determine the underlying disease.

## Figures and Tables

**Table 1:** Patient characteristics.

	All	GN	DKD	NS	*p*-Value
Number	90	30	25	35	
Sex (male/female)	56/34	13/17	20/5	23/12	0.017
Age, years	58.9 ± 18.3	49.9 ± 23.3	61.3 ± 10.8	64.7 ± 14.2	0.003
Height, cm	162.1 ± 8.9	162.0 ± 10.6	164.6 ± 7.6	160.5 ± 8.0	0.22
Body weight, kg	61.4 ± 13.1	59.4 ± 13.4	61.3 ± 10.8	63.0 ± 14.3	0.55
Body mass index, kg/m^2^	23.3 ± 4.4	22.6 ± 4.7	22.7 ± 3.7	24.3 ± 4.7	0.22
Diabetes mellitus, n (%)	38 (42.2)	1 (3.3)	25 (100)	12 (34.3)	<0.001
Hypertension, n (%)	61 (67.8)	11 (36.7)	22 (88.0)	28 (80.0)	<0.001
Serum Cr, mg/dL	1.01 (0.79–1.38)	0.82 (0.59–1.19)	1.14 (0.94–1.91)	1.08 (0.89–1.32)	0.010
eGFR, mL/min/1.73 m^2^	56.3 ± 27.9	68.4 ± 31.9	46.3 ± 24.4	53.2 ± 23.3	0.009
UPCR, g/gCr	1.41 (0.42–2.56)	0.68 (0.13–2.15)	2.85 (1.98–6.64)	1.15 (0.42–2.08)	<0.001
IFTA score	1.7 ± 1.0	1.3 ±1.0	2.0 ± 0.8	1.8 ± 1.0	0.015
uUMOD, mg/L	7.2 (3.7–24.5)	27.5 (7.2–44.0)	4.1 (1.7–7.2)	6.4 (3.2–14.8)	<0.001
uUMOD/Cr, mg/gCr	11.0 (5.5–22.6)	21.2 (10.3–31.3)	5.5 (3.6–12.3)	10.7 (6.4–19.9)	<0.001

GN, glomerulonephritis; DKD, diabetic kidney disease; NS, nephrosclerosis; eGFR, estimated glomerular filtration rate; UPCR, urinary protein-to-creatinine ratio; IFTA, interstitial fibrosis and tubular atrophy; uUMOD, urinary uromodulin; Cr, creatinine.

**Table 2:** Multivariate logistic regression analysis for the diagnosis of GN.

Variable	Odds Ratio	95% CI	*p*-Value
Age	0.963	0.93–1.00	0.051
Sex (male)	0.469	0.16–1.38	0.17
eGFR	0.988	0.96–1.02	0.47
Urinary protein-to-creatinine ratio	1.027	0.86–1.23	0.77
IFTA score	0.753	0.37–1.53	0.43
Urinary uromodulin-to-creatinine ratio	1.087	1.03–1.15	0.003

GN, glomerulonephritis; CI, confidence interval; eGFR, estimated glomerular filtration rate; IFTA, interstitial fibrosis and tubular atrophy.

## Data Availability

The datasets generated and/or analyzed during the current study are not publicly available because public data sharing was not included in the approved study protocol, but they are available from the corresponding author upon reasonable request.

## Abbreviations

The following abbreviations are used in this manuscript:

GN	Glomerulonephritis
DKD	Diabetic kidney disease
NS	Nephrosclerosis
uUMOD	Urinary uromodulin
uUMOD/Cr	Urinary uromodulin-to-creatinine ratio
eGFR	Estimated glomerular filtration rate
CKD	Chronic kidney disease
